# Association of pre-diagnostic physical exercise and peri-diagnostic body composition with mortality in non-metastatic colorectal cancer

**DOI:** 10.1007/s00384-023-04536-0

**Published:** 2023-09-27

**Authors:** David Renman, Bethany van Guelpen, Fredrick Anderson, Jan Axelsson, Katrine Riklund, Karin Strigård, Richard Palmqvist, Ulf Gunnarsson, Björn Gylling

**Affiliations:** 1 Department of Surgical and Perioperative Sciences, Surgery, Umeå University, Umeå, Sweden; 2 Department of Radiation Sciences, Oncology, Umeå University, Umeå, Sweden; 3 Wallenberg Centre for Molecular Medicine, Umeå University, Umeå, Sweden; 4 Department of Radiation Sciences, Radiation Physics, Umeå University, Umeå, Sweden; 5 Department of Radiation Sciences, Diagnostic Radiology, Umeå University, Umeå, Sweden; 6 Department of Medical Biosciences Pathology, Umeå University, Umeå, Sweden

**Keywords:** Colorectal cancer, Exercise, Myosteatosis, Physical activity, Sarcopenia

## Abstract

**Purpose:**

Sarcopenia and myosteatosis, quantified via computed tomography (CT), are associated with poor colorectal cancer outcomes. These body composition estimates can be influenced by physical exercise. We explored the correlation between pre-diagnostic physical exercise, body composition close to diagnosis, and the combined prognosis impact of these factors.

**Methods:**

We studied 519 stage I–III colorectal cancer (CRC) cases diagnosed 2000–2016 with pre-diagnostic self-reported recreational physical exercise data collected in the prospective, population-based Northern Sweden Health and Disease Study, and CT-estimated skeletal muscle index (SMI) or skeletal muscle density (SMD). Risk estimates were calculated by multivariable logistic regression and Cox proportional hazards models.

**Results:**

No association was seen between low pre-diagnostic physical exercise and sarcopenia/myosteatosis in the multivariable model adjusted for age, sex, educational level, tumor stage, and tumor location. In multivariable Cox regression models, the combination of low pre-diagnostic physical exercise and either sarcopenia or myosteatosis at the time of diagnosis was associated with cancer-specific mortality compared to the reference group of high physical exercise combined with no sarcopenia/myosteatosis (adjusted HR 1.94 95% CI 1.00–3.76 for sarcopenia and adjusted HR 2.39 95% CI 1.16–4.94 for myosteatosis).

**Conclusions:**

The combined presence of low pre-diagnostic physical exercise and sarcopenia or myosteatosis was associated with increased CRC-specific mortality. Despite the positive effect on prognosis, physical exercise did not alter body composition estimates at diagnosis, which could indicate attenuation from other factors.

**Supplementary Information:**

The online version contains supplementary material available at 10.1007/s00384-023-04536-0.

## Introduction

Pre-diagnostic physical activity is an important prognostic factor in colorectal cancer (CRC), reducing cancer-specific mortality by approximately 15% [1]. This may be due to a delay in the development of sarcopenia and/or myosteatosis [2, 3]. Sarcopenia is associated with mortality, functional decline, and a higher incidence of hospitalization [4].

In CRC patients, body composition measurements derived from computed tomography (CT) performed close to diagnosis have been associated with CRC mortality in numerous studies [5–7]. Two commonly used measurements are skeletal muscle index (SMI) and skeletal muscle density (SMD). Low SMI is often referred to as sarcopenia and low SMD as myosteatosis. Physical activity delays the onset of both sarcopenia [3] and myosteatosis [2]. Furthermore, physical activity in middle age is inversely associated with sarcopenia later in life [8]. Although resistance exercise increases muscle mass, strength and, quality [9], other and related potential underlying mechanisms by which physical activity prevents sarcopenia and myosteatosis are not fully understood, for example, prevention of age-related effects regarding insulin resistance, mitochondrial function, oxidative stress, and inflammation [9].

Body tissue composition is affected by the amount and type of physical activity [9] and can be measured by several methods. Both SMI and SMD can be standardized and calculated from CT scans at the level of the 3rd lumbar vertebra (L3). SMI correlates well with muscle mass in both healthy individuals [10] and cancer patients [11], while SMD correlates with skeletal muscle fat content in biopsies [12]. Both physical activity and body tissue composition are prognostic factors that can be evaluated using cohort and clinical data. However, although such analyses could provide novel insights into the role of physical activity in sarcopenia, myosteatosis, and prognosis in CRC patients, few studies have had access to data on physical activity/exercise. In particular, physical activity data collected before diagnosis, which reduces selection bias and recall bias, are rare in this type of setting. To the best of our knowledge, the interaction between pre-diagnostic physical activity or exercise and CT-estimated body composition in CRC patients, and their combined effect on cancer-specific mortality, has not been studied.

The aim of this study was to investigate the association between pre-diagnostic recreational physical exercise and sarcopenia or myosteatosis, respectively, in non-metastatic CRC patients, using data from a population-based prospective cohort. We also examined combinations of pre-diagnostic physical exercise and sarcopenia or myosteatosis in relation to overall and cancer-specific mortality.

## Methods

### Study population

This was a cohort study using retrospectively identified CRC patients from the prospective, population-based Västerbotten Intervention Programme (VIP). VIP, described in detail elsewhere [13], is an ongoing cardiometabolic health screening intervention, initiated over 30 years ago, to which inhabitants in Västerbotten County in Sweden are invited at a 10-year interval at ages 40, 50, and 60 years. VIP is a part of the umbrella cohort referred to as the Northern Sweden Health and Disease Study (NSHDS).

**Fig. 1:**
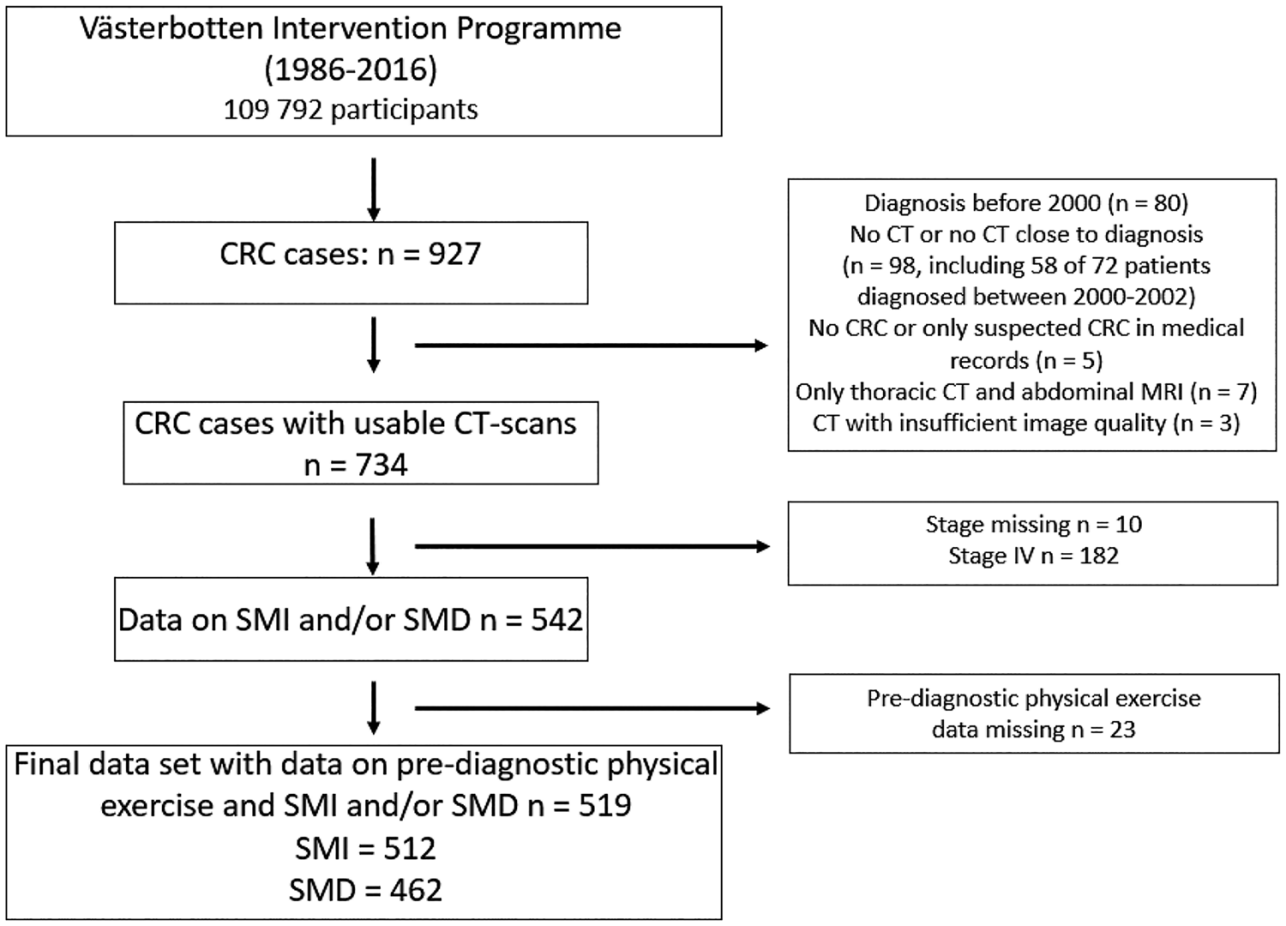
Flow chart of the study population and exclusions. CRC, colorectal cancer; CT, computed tomography; MRI, Magnetic resonance imaging; SMI, skeletal muscle index; SMD, skeletal muscle density

On the final recruitment date for this study, January 19, 2016, VIP included 109,792 participants (Fig. 1). CRC patients were identified using the Northern Sweden branch of the Swedish Cancer Registry (ICD-10 codes C18.0 and C18.2–18.9 for colon cancer and C19.9 and C20.9 for rectal cancer). Via medical records, clinical details were verified by a consultant pathologist specialized in gastrointestinal pathology (RP), including tumor location (right colon (caecum to the splenic flexure), left colon (splenic flexure to colon sigmoideum), or rectum (rectosigmoid junction and rectum)), and tumor stage at diagnosis.

A total of 927 CRC cases with pre-diagnostic participation in VIP were identified. Of these, usable CT-scans and data on SMI and/or SMD were available in 734 cases. Exclusions were made due to no CT or no CT close to diagnosis (*n* = 98), no CRC or only suspected CRC in medical records (*n* = 5), only thoracic CT-scan and abdominal magnetic resonance imaging (MRI) (*n* = 7), and insufficient image quality (*n* = 3). Storage of CT images began 2000; therefore, all patients diagnosed before 2000 were excluded (*n* = 80). The digital storage was gradually implemented in the years 2000–2002, and most patients (*n* = 58 of 72) without digital CT-scan missing were excluded. Of cases with data on SMI or SMD exclusion were made for stage IV CRC (*n* = 182) or missing stage (*n* = 10) and missing data on physical exercise (*n* = 23). This resulted in a final data set of 519 observations (Fig. 1). Cancer-specific mortality was defined as death with known disseminated or recurrent disease, and cases were censored at the end of follow-up or death from other causes, as judged by a consulting colorectal surgeon using the Northern Sweden Cancer Registry and individual patient records. Overall mortality was defined as death from any cause. Patients were followed until the time of death, censoring, or the end of the study (May 2, 2022). Two patients (*n* = 2) were excluded from the survival analysis due to death within 30 days after diagnosis to account for deaths due to perioperative complications.

### Variables

The self-reported recreational physical exercise variable was collected from a single questionnaire item in VIP phrased “How often have you exercised in sportswear over the past three months, with the intent to improve your fitness and/or well-being?”. Answers were reported on a five-level scale (“never,” “now and then,” “1–2 times per week,” “2–3 times per week,” and “ > 3 times per week”). We classified the two categories “never” and “now and then” as “low physical exercise,” and physical exercise one or more times per week as “high physical exercise.” Additional variables collected from the VIP were self-reported education status (no secondary-, secondary-, or post-secondary education), self-reported tobacco smoking (never, former-, current smoker), and diabetes (yes/no). Participants were defined as diabetic based on self-report or fasting blood glucose level of > 7 mmol/l and/or glucose level > 12.2 mmol/l 2 h after glucose administration in an oral glucose tolerance test using 75 g glucose load according to the World Health Organization’s (WHO) criteria [14]. When a participant had two or more observations in the VIP prior to CRC diagnosis, the observation with the shortest time from observation to diagnosis was used.

### Definition of sarcopenia and myosteatosis

We used cut-offs for SMI and SMD from a previous study on the same cohort [15]. In short, the most caudal slice of a CT scan with both transverse processes of the L3-vertebra visible was used to measure the cross-sectional area of fat and muscle in cm^2^. SMD was measured as the mean Hounsfield Unit (HU) across the delineated muscle area and SMI was measured as the total muscle area in cm^2^ adjusted for height squared (m^2^). Two of the authors (BG and FA) conducted the measurements under supervision of a senior consultant radiologist (KR). The CT scans were performed within 4 months before and 3 months after diagnosis, with the majority (91%) within 1 month of diagnosis. Nineteen patients (3.7% for SMI and 4.1% for SMD) had their CT scan performed after surgery.

We defined sarcopenia as the lowest sex-specific tertile of SMI (43.1 cm^2^/m^2^ for men and 32.9 cm^2^/m^2^ for women) and myosteatosis as the lowest sex-specific tertile of SMD (38.5 HU for men and 36.1 HU for women). There is no scientific consensus on what cut-off level to use, and muscle index and attenuation can vary between populations as can the measurement methods. In a recent meta-analysis of 44 studies on CT-estimated sarcopenia in CRC [5], 21 different definitions of sarcopenia were used, including optimal stratification, quartiles, tertiles, and sex-specific cut-offs. In a similar meta-analysis on myosteatosis [6], there were 19 different definitions of myosteatosis in the 40 studies included. Therefore, we used cut-offs defined based on the lowest tertile of SMI and SMD in our previous study, in which sarcopenia and myosteatosis estimates showed associations with mortality similar to a large meta-analysis [15].

### Statistical analysis

Associations between physical exercise and body composition measures were estimated using logistic regression, with physical exercise as the independent variable and SMI or SMD as the dependent variable. Multivariable models were used to adjust for potential confounders including age at diagnosis (linear), tumor location, tumor stage (I, II, and III), sex (male or female), as well as educational level (which significantly differed between physical exercise groups (Table 1)), due to their possible roles as confounders of associations between the exposure and outcome variables [3–5, 15, 16]. In four patients, data points on educational level were missing and were imputed using sex-specific mode.

**Table 1:** Characteristics of the study population

	Recreational physical exercise		
	Total	Low physical exercise (*n* = 373)	High physical exercise (*n* = 146)
Sex, *n* (%)			
Man	256	194 (52.0)	69 (47.3)
Woman	263	179 (48.0)	77 (52.7)
Age at diagnosis	519	67.9 (61.5–73.5)^a^	65.5 (58.1–72.0)^a^
Time from cohort participation to diagnosis in years	519	10.4 (6.4–15.1)^a^	7.8 (4.7–13.5)^a^
Age at cohort participation^c^, *n* (%)			
40 years	42	23 (8.5)	5 (5.1)
50 years	117	79 (29.2)	26 (32.9)
60 years	360	169 (62.4)	49 (62.0)
Tumor site^b^, *n* (%)			
Right	163	125 (33.5)	38 (26.0)
Left	159	118 (31.6)	41 (28.1)
Rectal	197	130 (34.9)	67 (45.9)
Stage^b^, *n* (%)			
I	137	100 (26.8)	37 (25.3)
II	196	147 (39.4)	49 (33.6)
III	186	126 (33.8)	60 (41.1)
Smoking status^c^, *n* (%)			
Never	217	155 (42.2)	62 (43.4)
Former	198	138 (37.6)	60 (42.0)
Current	95	74 (20.2)	21 (14.7)
Missing	9	6	3
Diabetes^c^, *n* (%)			
No	464	335 (90.1)	129 (89.0)
Yes	53	37 (10.0)	16 (11.0)
Missing	2	1	1
Education status^c^, *n* (%)			
No secondary	336	261 (70.4)	75 (52.1)
Secondary	87	56 (15.1)	31 (21.5)
Post-secondary	92	54 (14.6)	38 (26.4)
Missing	4	2	2

^a^Results displayed as median (25–75 percentile)

^b^Variable collected after the diagnosis

^c^Variable collected at the time of participation in the Västerbotten Invervention Programme

Characteristics of 519 non-metastatic colorectal cancer patients, according to self-reported pre-diagnostic recreational physical exercise

Uni- and multivariable Cox proportional hazard models were used for survival analyses. The covariates included were the same as for the logistic regression analyses. These models were applied for overall and cancer-specific mortality for both sarcopenia and myosteatosis. The proportional odds assumption was checked using Schoenfeldt’s residual-based test for each multivariable model. When the assumption was violated, the model was stratified for the variable or variables that violated the assumption. Stratified models did not violate the proportional hazard assumption and are presented as the final results. Log-likelihood test was conducted to compare the multivariable model including physical exercise, sarcopenia/myosteatosis, covariates (age at diagnosis, tumor location, tumor stage, sex and education level), and an interaction term for physical exercise and sarcopenia/myosteatosis to the same model without the interaction term. This was done for both cancer-specific and overall mortality for both sarcopenia and myosteatosis. *p* values for the log-likelihood test of interaction are displayed in Tables 3 and 4.

In all survival models, we calculated mortality for pre-diagnostic physical exercise level, sarcopenia/myosteatosis, as well as in an additive interaction model using a cross-classified variable combining pre-diagnostic physical exercise level and sarcopenia/myosteatosis status (divided into four categories: high physical exercise and non-sarcopenic/non-myosteatotic, high physical exercise and sarcopenic/myosteatotic, low physical exercise and non-sarcopenic/non-myosteatotic, and low physical exercise and sarcopenic/myosteatotic). The starting date in the survival analysis was the date of diagnosis. We also conducted causal mediation analyses testing sarcopenia and myosteatosis as potential mediators of the relationship between physical exercise and both overall and cancer-specific mortality.

To account for reverse causality, i.e., possible changes in exercise habits due to sarcopenia or cancer symptoms in the period approaching colorectal cancer diagnosis, we conducted sensitivity analyses excluding cases diagnosed within one year after participation in VIP (*n* = 19). Sensitivity analyses were also done excluding patients with CT scans after surgery (*n* = 19), to account for any effects of surgery on body composition.

All statistical calculations were made using STATA version 18 (StataCorp LP).

This study was approved by the Regional Ethics Review Board in Umeå, Sweden (Dnr 2016–221-31) and the Swedish Ethical Review Authority (Dnr 2019–00157).

## Results

The final study population included 519 CRC patients with data on sarcopenia, myosteatosis, or both. The proportions of patients with sarcopenia and myosteatosis were 30.7% and 31.4%, respectively. The proportion of women was 49.3%, and the mean age at diagnosis was 66.5 years (range 41.5–85.6). Patients with high pre-diagnostic physical exercise were younger at diagnosis, had a shorter time between participation and diagnosis, and had a higher educational level compared to the low physical exercise group (Table 1). The median time from reporting of physical exercise to diagnosis was 9.6 years (IQR 5.8–14.8). The median time from diagnosis until the end of the study period for patients included in the survival analysis and alive at follow-up was 10.2 years (IQR 7.8–13.6 years, *n* = 330) for the sarcopenia models and 10.2 years (IQR 7.7–13.7 years, *n* = 301) for the myosteatosis models.

Low pre-diagnostic physical exercise was not associated with either sarcopenia or myosteatosis in the multivariable logistic regression model, in which the odds ratio (OR) for sarcopenia was attenuated and no longer statistically significant compared to the univariable analysis (adjusted OR 1.37, 95% CI 0.86–2.19, Table 2).

**Table 2:** Pre-diagnostic recreational physical exercise in relation to peri-diagnostic sarcopenia and myosteatosis in non-metastatic colorectal cancer

		Univariable^a^			Multivariable^b^		
	N	Low physical exercise	High physical exercise	*p* value	Low physical exercise	High physical exercise	*p* value
Sarcopenia^c^	512	1.62 (1.04–2.52)	Ref 1.0	0.031	1.37 (0.86–2.19)	Ref 1.0	0.184
Myosteatosis^c^	462	1.20 (0.77–1.86)	Ref 1.0	0.418	0.95 (0.59–1.51)	Ref 1.0	0.819

^a^Univariable logistic regression analysis with physical exercise level as the independent variable and sarcopenia or myosteatosis as the dependent variable

^b^Multivariable logistic regression analysis. Variables adjusted for in the multivariable models are stage, tumor location, age at diagnosis, sex, and education level

^c^Results displayed as odds ratio (95% confidence interval)

In the analyses of overall and cancer-specific mortality, sarcopenia, myosteatosis, and low physical exercise generally demonstrated HRs above one but not statistically significant. Exceptions were sarcopenia, and overall mortality (adjusted HR 1.49 95% CI 1.09–2.04) and null associations for low physical exercise and overall mortality. (Tables 3 and 4). For both sarcopenia and myosteatosis, combination with low physical exercise was associated with higher cancer-specific mortality compared to their respective reference category of no sarcopenia/myosteatosis and high physical exercise (adjusted HR 1.94 95% CI 1.00–3.76 for sarcopenia and low physical exercise, adjusted HR 2.39 95% CI 1.16–4.94 for myosteatosis and low physical exercise). For other combination categories, HRs were lower and non-significant. For overall mortality, only the HR for the combination of myosteatosis and high physical exercise was statistically significant (adjusted HR 2.08 95% CI 1.12–3.88) (Tables 3 and 4). In causal mediation analyses, neither sarcopenia nor myosteatosis mediated the relationship between physical exercise and overall or cancer-specific survival, with indirect effects close to zero for all analyses (Supplementary Table 1).

**Table 3:** Pre-diagnostic recreational physical exercise and peri-diagnostic sarcopenia

	Overall mortality			
	*N* = 510 (%)	Univariable^a^	Multivariable^acd^	*p* value
Sarcopenia				
Yes	156 (31)	1.65 (1.22–2.23)	1.49 (1.09–2.04)	0.012
No	354 (69)	Ref	Ref	Ref
Physical exercise				
Low	367 (72)	1.16 (0.83–1.61)	1.03 (0.73–1.45)	0.888
High	143 (28)	Ref	Ref	Ref
Sarcopenia and exercise combined^e^				
No sarcopenia + High exercise	109 (21)	Ref	Ref	Ref
Sarcopenia + High exercise	34 (7)	1.81 (0.98–3.34)	1.56 (0.83–2.92)	0.167
No sarcopenia + Low exercise	245 (48)	1.14 (0.76–1.72)	1.02 (0.67–1.55)	0.936
Sarcopenia + low exercise	122 (24)	1.81 (1.17–2.81)	1.50 (0.95–2.37)	0.085

	Cancer-specific mortality			
	N = 510 (%)	Univariable^a^	Multivariable^abd^	*p* value
Sarcopenia				
Yes	156 (31)	1.22 (0.80–1.85)	1.40 (0.90–2.16)	0.136
No	354 (69)	Ref	Ref	Ref
Physical exercise				
Low	367 (72)	1.36 (0.85–2.17)	1.40 (0.86–2.28)	0.179
High	143 (28)	Ref	Ref	Ref
Sarcopenia and exercise combined^f^				
No Sarcopenia + High exercise	109 (21)	Ref	Ref	Ref
Sarcopenia + high exercise	34 (7)	1.72 (0.71–4.18)	2.05 (0.82–5.13)	0.124
No sarcopenia + Low exercise	245 (48)	1.52 (0.87–2.67)	1.58 (0.88–2.84)	0.125
Sarcopenia + low exercise	122 (24)	1.64 (0.88–3.05)	1.94 (1.00–3.76)	0.048

^a^Results displayed as hazard ratios (95% confidence interval)

^b^Analysis stratified for tumor location and education level

^c^Analysis stratified for tumor location

^d^Variables adjusted for in the multivariable models are stage, tumor location, age at diagnosis, sex, and education level

^e^*p* value = 0.877 for log-likelihood test comparing the multivariable model including sarcopenia, physical exercise, covariates, and the interaction between sarcopenia and physical exercise to the multivariable model without the interaction term

^f^*p* value = 0.337 for log-likelihood test comparing the multivariable model including sarcopenia, physical exercise, covariates, and the interaction between sarcopenia and physical exercise to the multivariable model without the interaction term

Pre-diagnostic physical exercise and peri-diagnostic sarcopenia in relation to overall and cancer-specific mortality in non-metastatic colorectal cancer

**Table 4:** Pre-diagnostic recreational physical exercise and peri-diagnostic myosteatosis

	Overall mortality			
	*N* = 460 (%)	Univariable^a^	Multivariable^ac^	*p* value
Myosteatosis				
Yes	143 (31)	1.75 (1.28–2.40)	1.38 (0.99–1.92)	0.057
No	317 (69)	Ref	Ref	Ref
Physical exercise				
Low	325 (71)	1.18 (0.83–1.68)	1.07 (0.74–1.54)	0.726
High	135 (29)	Ref	Ref	Ref
Myosteatosis and exercise combined^d^				
No myosteatosis + high exercise	97 (21)	Ref	Ref	Ref
Myosteatosis + high exercise	38 (8)	2.82 (1.55–5.14)	2.08 (1.12–3.88)	0.021
No myosteatosis + low exercise	220 (48)	1.47 (0.92–2.36)	1.32 (0.82–2.14)	0.253
Myosteatosis + low exercise	105 (23)	2.15 (1.30–3.53)	1.58 (0.93–2.66)	0.090

	Cancer-specific mortality			
	*N* = 460 (%)	Univariable^a^	Multivariable^abc^	*p* value
Myosteatosis				
Yes	143 (31)	1.49 (0.97–2.30)	1.46 (0.92–2.31)	0.104
No	317 (69)	Ref	Ref	Ref
Physical exercise				
Low	325 (71)	1.48 (0.90–2.44)	1.58 (0.94–2.64)	0.085
High	135 (29)	Ref	Ref	Ref
Myosteatosis and exercise combined^e^				
No myosteatosis + high exercise	97 (21)	Ref	Ref	Ref
Myosteatosis + high exercise	38 (8)	2.04 (0.83–4.99)	1.72 (0.68–4.34)	0.250
No myosteatosis + low exercise	220 (48)	1.69 (0.89–3.20)	1.71 (0.89–3.28)	0.106
Myosteatosis + low exercise	105 (23)	2.24 (1.13–4.43)	2.39 (1.16–4.94)	0.019

^a^Results displayed as hazard ratios (95% confidence interval)

^b^Analysis stratified for tumor location

^c^Variables adjusted for in the multivariable are stage, tumor location, age at diagnosis, sex, and education level

^d^*p* value = 0.128 for log-likelihood test comparing the multivariable model including myosteatosis, physical exercise, covariates, and the interaction between myosteatosis and physical exercise to the multivariable model without the interaction term

^e^*p* value = 0.694 for log-likelihood test comparing the multivariable model including myosteatosis, physical exercise, covariates, and the interaction between myosteatosis and physical exercise to the multivariable model without the interaction term

Pre-diagnostic recreational physical exercise and peri-diagnostic myosteatosis in relation to overall and cancer-specific mortality in non-metastatic colorectal cancer

Sensitivity analyses excluding patients with less than 1 year between participation in the VIP cohort (physical exercise reporting) and CRC diagnosis did not affect the main results (Supplementary Tables 2 and 3). Neither did sensitivity analysis excluding patients whose CT scan was performed after surgery (Supplementary Tables 4 and 5).

## Discussion

In this study on 519 non-metastatic CRC patients from a population-based cohort, the combination of low pre-diagnostic physical exercise and the presence of either sarcopenia or myosteatosis was associated with increased cancer-specific mortality in multivariable Cox regression analyses. These results were congruent with our hypothesis that low pre-diagnostic physical exercise combined with sarcopenia or myosteatosis have an additive effect on cancer-specific mortality.

In our data, low pre-diagnostic physical exercise was associated with the presence of sarcopenia in the univariable model. However, the association was attenuated after adjusting for age, sex, educational level, tumor stage, and location (Table 2). Previous studies indicate that exercise in middle age prevents the development of sarcopenia in healthy individuals [8] and that physical activity delays the onset of both sarcopenia [3] and myosteatosis [2]. This association has not been investigated in a CRC population until now. A previous study on physical activity levels across adult life showed that participants in the highest tertile of recreational physical activity had stronger grip strength when measured in older age compared to those in the lowest tertile [17]. No association was seen in the middle tertile in that study. In the present study, we dichotomized the groups into low or high pre-diagnostic physical exercise. This could potentially result in some participants in the high physical exercise group not being sufficiently active to affect the development of sarcopenia and myosteatosis. On the other hand, using the original five-level scale would have affected the power of the statistical calculations negatively because of small groups with little actual difference in physical exercise.

The Cox proportional hazard analysis on cancer-specific mortality showed a significantly increased HR for both combinations of low pre-diagnostic physical exercise with sarcopenia or myosteatosis in the multivariable model. This pattern was not evident in the overall mortality models. In the cancer-specific models, there are fewer events, but those events are related to CRC. If there is a true additive effect on tumor progression, it would therefore be more likely to be apparent in the cancer-specific mortality models, as suggested from our results. However, the risk estimates did not provide definitive evidence of an interaction.

The cause of sarcopenia is multifactorial and can be due to age-related loss of muscle mass, or be secondary to systemic disease, inflammatory changes, or physical inactivity [16]. In older individuals with sarcopenia, serum growth hormone (GH) levels and insulin-like growth factor (IGF-1) levels are decreased [18]. Other factors related to sarcopenia are increased mitochondrial damage leading to increased levels of reactive oxygen species (ROS), increased inflammation, and impaired regeneration of skeletal muscle [19]. Similarly, physical activity increases serum levels of GH and IGF-1 [20] as well as stimulates mitochondrial biogenesis[21] and is beneficial for immunological health [22]. Thus, low physical activity and sarcopenia seem to affect our bodies in a similar manner. This could potentially explain the additive effect in the present study.

To our knowledge, no previous study has investigated the combined effect of physical exercise levels and the presence of sarcopenia or myosteatosis on CRC mortality based on prospectively collected data. In previously published meta-analyses on the effect of sarcopenia or myosteatosis on mortality in CRC [5–7], only two of 53 studies had data on physical activity/exercise [23, 24]. In both studies, these data were derived from preoperative post-diagnostic physical tests, at which point the participants may be sarcopenic or myosteatotic due to reduced physical activity/exercise and/or nutritional intake in the symptomatic period prior to diagnosis or tumor-induced cachexia. In our study, we used data on physical exercise collected well before the diagnosis (median 9.6 years), representing habitual physical exercise over the long term. This novel perspective broadens the knowledge on the interplay between physical exercise and body tissue composition in CRC.

Self-reported data on physical activity/exercise is commonly used in epidemiological studies with good reliability, albeit with a tendency to underestimate sedentary time [25]. The physical activity questionnaire in VIP has been included in a multicenter validation including the Cambridge Index [26] but has not been individually validated. Other types of physical activity, such as occupational activity and commuting habits, are also explored in the VIP questionnaire. However, these measures, which are not validated and for which the phrasing of the questions have varied in over time, were not used in our analysis. Instead, we selected physical exercise as the most reliable indicator of strenuous physical activity among participants. To adjust for potential changes in physical exercise that might be linked to undiagnosed CRC, we performed a sensitivity analysis that excluded patients who participated in VIP within 1 year of diagnosis. This analysis did not significantly alter the results. Our study was limited by a lack of repeated physical exercise data and objective measures of physical performance status. However, the prospective recording of physical activity mitigates the risk of recall bias. There were some significant differences between the exercise groups, which might have affected our findings. The high physical exercise group was younger at diagnosis, hypothetically due to a higher proportion of rectal cancer, which is commonly associated with younger age at diagnosis [27].

Both sarcopenia and low physical activity are linked with diseases such as diabetes, dementia, and arteriosclerosis [28–30]. In our study, we only had access to data on diabetes at the time of participation in the Västerbotten Intervention Programme, which is a limitation since subsequent diagnosis of diabetes and other comorbidities could also affect mortality. This was partially accounted for by analyzing both cancer-specific and overall mortality. Another limitation was the lack of cancer therapy data. However, since we adjusted for both tumor stage and location, and excluded the heterogeneously treated group of stage IV CRC patients, the effect on the results should be minimal [31]. Another limitation with this study was the lack of other clinical outcome variables such as length of hospital stay, postoperative complications, re-operations, operation time, or re-admissions. These might be relevant endpoints but by adjusting for both tumor stage and location as well as exclusion of stage IV disease partially accounted for this. Finally, our results cannot be used for prognostic algorithms or other practical application, but this was also not our intent as the focus was to gain insights into the potential etiological role of physical exercise in sarcopenia and myosteatosis in CRC and to explore the possibility of synergistic effects on mortality.

We used prospectively collected physical exercise data from a population-based cohort with a high participation rate [13] which is a strength and reduces the risk of reverse causality and recall bias. Other strengths of this study were the relatively large sample size, though limited for some analyses, the long follow-up time after diagnosis in the survival analyses, as well as the availability of data on both overall and cancer-specific mortality.

## Conclusions

In this study on data from a population-based cohort, we saw no association between low pre-diagnostic physical exercise and CT-estimated sarcopenia or myosteatosis in non-metastatic CRC. The combined presence of low pre-diagnostic physical exercise and sarcopenia or myosteatosis was associated with increased CRC-specific mortality, but given the limited sample size in the analysis, additional data will be required to determine whether this finding reflects an additive detrimental effect.

### Supplementary Information

Below is the link to the electronic supplementary material.

Supplementary file1 (DOCX 14 KB)

Supplementary file2 (DOCX 14 KB)

Supplementary file3 (DOCX 18 KB)

Supplementary file4 (DOCX 14 KB)

Supplementary file5 (DOCX 18 KB)

## Data Availability

The datasets used and analyzed during the current study are available from the corresponding author on reasonable request.

## List of abbreviations

CI: Confidence interval
CRC: Colorectal cancer
CT: Computed tomography
GH: Growth hormone
HU: Hounsfield units
HR: Hazard ratio
IGF: Insulin-like growth factor
NSHDS: Northern Sweden Health and Disease Study
OR: Odds ratio
SMD: Skeletal muscle density
SMI: Skeletal muscle index
VIP: Västerbotten intervention programme
WHO: World Health Organization
