# Supplementary material for: Association of pre-diagnostic physical exercise and peri-diagnostic body composition with mortality in non-metastatic colorectal cancer
Source: Int J Colorectal Dis. 2023 Sep 27;38(1):239. doi: 10.1007/s00384-023-04536-0 (PMC10533590; doi:10.1007/s00384-023-04536-0)
Supplement: Supplementary file 1 — Supplementary file1 (DOCX 14 KB) [file 384_2023_4536_MOESM1_ESM.docx]

|  | Overall Mortality | | |
| --- | --- | --- | --- |
|  | Total Effect (TE) | Indirect Effect (IE) | Direct Effect (DE) |
| Sarcopenia  Myosteatosis | -0.03 (-0.13 – 0.06) | -0.01 (-0.04 – 0.01) | -0.02 (-0.11 – 0.07) |
|  | -0.04 (-0.13 – 0.06) | -0.01 (-0.04 – 0.12) | -0.03 (-0.12 – 0.07) |
|  | Cancer-Specific Mortality | | |
|  | Total Effect (TE) | Indirect Effect (IE) | Direct Effect (DE) |
| Sarcopenia  Myosteatosis | -0.05 (-0.12 – 0.02) | -0.01 (-0.02 – 0.01) | -0.04 (-0.12 – 0.03) |
|  | -0.06 (-0.14 – 0.01) | -0.00 (-0.01 – 0.01) | -0.06 (-0.13 – 0.02) |

## Supplementary Table 1

**Supplementary Table 1.** Causal mediation analyses for mediation effect from pre-diagnostic physical exercise on mortality through sarcopenia and myosteatosis for both overall and cancer-specific mortality.
