# Supplementary material for: Association of pre-diagnostic physical exercise and peri-diagnostic body composition with mortality in non-metastatic colorectal cancer
Source: Int J Colorectal Dis. 2023 Sep 27;38(1):239. doi: 10.1007/s00384-023-04536-0 (PMC10533590; doi:10.1007/s00384-023-04536-0)
Supplement: Supplementary file 2 — Supplementary file2 (DOCX 14 KB) [file 384_2023_4536_MOESM2_ESM.docx]

## Supplementary Table 2

**Supplementary Table 2.** Sensitivity analysis for pre-diagnostic recreational physical exercise and peri-diagnostic sarcopenia and myosteatosis in non-metastatic colorectal cancer, excluding patients with physical exercise data collected within one year prior to diagnosis.

|  |  | Univariable^a^ | | | Multivariable^b^ | | |
| --- | --- | --- | --- | --- | --- | --- | --- |
|  | N | Low Physical Exercise | High Physical Exercise | p-value | Low Physical Excercise | High Physical Exercise | p-value |
| Sarcopenia^c^ | 493 | 1.60 (1.02-2.50) | Ref 1.0 | 0.041 | 1.39 (0.87-2.24) | Ref 1.0 | 0.171 |
| Myosteatosis^c^ | 444 | 1.16 (0.74-1.81) | Ref 1.0 | 0.514 | 0.95 (0.59-1.54) | Ref 1.0 | 0.849 |

^a^Univariable logistic regression analysis with physical exercise level as independent variable and sarcopenia or myosteatosis as dependent variable.
^b^Multivariable logistic regression analysis. Variables adjusted for in the multivariable models are stage, tumor location, age at diagnosis, sex and education level.
^c^Results displayed as Odds ratio (95% confidence interval)
