# Supplementary material for: Association of pre-diagnostic physical exercise and peri-diagnostic body composition with mortality in non-metastatic colorectal cancer
Source: Int J Colorectal Dis. 2023 Sep 27;38(1):239. doi: 10.1007/s00384-023-04536-0 (PMC10533590; doi:10.1007/s00384-023-04536-0)
Supplement: Supplementary file 3 — Supplementary file3 (DOCX 18 KB) [file 384_2023_4536_MOESM3_ESM.docx]

## Supplementary Table 3

| Sarcopenia Cancer-Specific Mortality^a^ | | |  |  |  |
| --- | --- | --- | --- | --- | --- |
| N = 491 | Univariable | Multivariable^bc^ | p-value |  |  |
| Sarcopenia  Yes  No | 1.27 (0.83-1.93)  Ref | 1.43 (0.92-2.22)  Ref | 0.110  Ref |  |  |
| Physical Exercise  Low  High | 1.38 (0.85-2.24)  Ref | 1.45 (0.88-2.39)  Ref | 0.147  Ref |  |  |
| Sarcopenia and Exercise combined  No Sarcopenia + High Exercise  Sarcopenia + High Exercise  No Sarcopenia + Low Exercise  Sarcopenia + Low Exercise | Ref  1.85 (0.75-4.58)  1.57 (0.87-2.85)  1.74 (0.91-3.33) | Ref  2.10 (0.83-5.31)  1.64 (0.89-3.01)  2.06 (1.05-4.05) | Ref  0.117  0.111  0.036 |  |  |
| Myosteatosis Cancer-Specific Mortality^a^ | | |  |  |  |
| N = 442 | Univariable | Multivariable^c^ | p-value |  |  |
| Myosteatosis  Yes  No | 1.50 (0.97-2.33)  Ref | 1.43 (0.90-2.27)  Ref | 0.134  Ref |  |  |
| Physical Exercise  Low  High | 1.51 (0.90-2.54)  Ref | 1.65 (0.96-2.83)  Ref | 0.067  Ref |  |  |
| Myosteatosis and Exercise combined  No Myosteatosis + High Exercise  Myosteatosis + High Exercise  No Myosteatosis + Low Exercise  Myosteatosis + Low Exercise | | Ref  2.33 (0.92-5.90)  1.85 (0.92-3.69)  2.38 (1.14-4.96) | Ref  1.94 (0.74-5.08)  1.93 (0.96-3.89)  2.54 (1.18-5.49) | Ref  0.178  0.066  0.018 |  |

Sensitivity analysis for pre-diagnostic recreational physical activity and peri-diagnostic sarcopenia and myosteatosis related to cancer-specific mortality in non-metastatic colorectal cancer, excluding patients with physical activity data collected within one year prior to diagnosis.

^a^ Results displayed as Hazard Ratios (95% Confidence interval)
^b^Analysis stratified for tumor location.
^c^Variables adjusted for in the multivariable models are stage, tumor location, age at diagnosis, sex and education level.
