# Supplementary material for: Association of pre-diagnostic physical exercise and peri-diagnostic body composition with mortality in non-metastatic colorectal cancer
Source: Int J Colorectal Dis. 2023 Sep 27;38(1):239. doi: 10.1007/s00384-023-04536-0 (PMC10533590; doi:10.1007/s00384-023-04536-0)
Supplement: Supplementary file 4 — Supplementary file4 (DOCX 14 KB) [file 384_2023_4536_MOESM4_ESM.docx]

## Supplementary Table 4

**Supplementary Table 4.** Sensitivity analysis for pre-diagnostic recreational physical exercise and peri-diagnostic sarcopenia and myosteatosis in non-metastatic colorectal cancer, excluding patients with CT scan performed after surgery.

|  |  | Univariable^a^ | | | Multivariable^b^ | | |
| --- | --- | --- | --- | --- | --- | --- | --- |
|  | N | Low Physical Exercise | High Physical Exercise | p-value | Low Physical Exercise | High Physical Exercise | p-value |
| Sarcopenia^c^ | 493 | 1.67 (1.06-2.62) | Ref 1.0 | 0.027 | 1.43 (0.89-2.32) | Ref 1.0 | 0.143 |
| Myosteatosis^c^ | 443 | 1.16 (0.74-1.82) | Ref 1.0 | 0.516 | 0.93 (0.58-1.51) | Ref 1.0 | 0.777 |

^a^Univariable logistic regression analysis with physical exercise level as independent variable and sarcopenia or myosteatosis as dependent variable.
^b^Multivariable logistic regression analysis. Variables adjusted for in the multivariable models are stage, tumor location, age at diagnosis, sex and education level.
^c^Results displayed as Odds ratio (95% confidence interval)
