# Supplementary material for: Association of pre-diagnostic physical exercise and peri-diagnostic body composition with mortality in non-metastatic colorectal cancer
Source: Int J Colorectal Dis. 2023 Sep 27;38(1):239. doi: 10.1007/s00384-023-04536-0 (PMC10533590; doi:10.1007/s00384-023-04536-0)
Supplement: Supplementary file 5 — Supplementary file5 (DOCX 18 KB) [file 384_2023_4536_MOESM5_ESM.docx]

## Supplementary Table 5

| Sarcopenia Cancer-Specific Mortality^a^ | | |  |
| --- | --- | --- | --- |
| N = 491 | Univariable | Multivariable^bc^ | p-value |
| Sarcopenia  Yes  No | 1.24 (0.81-1.89)  Ref | 1.32 (0.85-2.06)  Ref | 0.214  Ref |
| Physical Exercise  Low  High | 1.32 (0.82-2.12)  Ref | 1.39 (0.85-2.29)  Ref | 0.189  Ref |
| Sarcopenia and Exercise combined  No Sarcopenia + High Exercise  Sarcopenia + High Exercise  No Sarcopenia + Low Exercise  Sarcopenia + Low Exercise | Ref  1.45 (0.57-3.71)  1.37 (0.78-2.42)  1.57 (0.84-2.94) | Ref  1.48 (0.57-3.84)  1.43 (0.80-2.57)  1.79 (0.93-3.45) | Ref  0.426  0.231  0.081 |
| Myosteatosis Cancer-Specific Mortality^a^ | | |  |
| N = 442 | Univariable | Multivariable^c^ | p-value |
| Myosteatosis  Yes  No | 1.42 (0.91-2.21)  Ref | 1.37 (0.85-2.20)  Ref | 0.194  Ref |
| Physical Exercise  Low  High | 1.43 (0.86-2.39)  Ref | 1.58 (0.93-2.69)  Ref | 0.092  Ref |
| Myosteatosis and Exercise combined  No Myosteatosis + High Exercise  Myosteatosis + High Exercise  No Myosteatosis + Low Exercise  Myosteatosis + Low Exercise | Ref  1.69 (0.67-4.30)  1.54 (0.81-2.93)  2.02 (1.01-4.05) | Ref  1.42 (0.54-3.71)  1.62 (0.84-3.11)  2.21 (1.06-4.62) | Ref  0.474  0.151  0.035 |

**Supplementary Table 5.** Sensitivity analysis for pre-diagnostic recreational physical exercise and peri-diagnostic sarcopenia and myosteatosis related to cancer-specific mortality in non-metastatic colorectal cancer, excluding patients with CT scan performed after surgery.

^a^ Results displayed as Hazard Ratios (95% Confidence interval)
^b^Analysis stratified for tumor location.
^c^Variables adjusted for in the multivariable models are stage, tumor location, age at diagnosis, sex and education level.
